# Indoor Air Quality in Domestic Environments during Periods Close to Italian COVID-19 Lockdown

**DOI:** 10.3390/ijerph18084060

**Published:** 2021-04-12

**Authors:** Maria Chiara Pietrogrande, Lucia Casari, Giorgia Demaria, Mara Russo

**Affiliations:** Department of Chemical, Pharmaceutical and Agricultural Sciences, University of Ferrara, Via Fossato di Mortara 17/19, 44121 Ferrara, Italy; lucia.casari@unife.it (L.C.); dmrgrg@unife.it (G.D.); rssmra1@unife.it (M.R.)

**Keywords:** indoor air quality, dwellings, PM_2.5_, VOCs, CO_2_ concentration, low-cost IAQ sensors, COVID-19

## Abstract

This paper describes the in situ monitoring of indoor air quality (IAQ) in two dwellings, using low-cost IAQ sensors to provide high-density temporal and spatial data. IAQ measurements were conducted over 2-week periods in the kitchen and bedroom of each home during the winter, spring, and summer seasons, characterized by different outside parameters, that were simultaneously measured. The mean indoor PM_2.5_ concentrations were about 15 μg m^−3^ in winter, they dropped to values close to 10 μg m^−3^ in spring and increased to levels of about 13 μg m^−3^ in summer. During the winter campaign, indoor PM_2.5_ was found mainly associated with particle penetration inside the rooms from outdoors, because of the high outdoor PM_2.5_ levels in the season. Such pollution winter episodes occur frequently in the study region, due to the combined contributions of strong anthropogenic emissions and stable atmospheric conditions. The concentrations of indoor volatile organic compounds (VOCs) and CO_2_ increased with the number of occupants (humans and pets), as likely associated with consequent higher emissions through breathing and metabolic processes. They also varied with occupants’ daily activities, like cooking and cleaning. Critic CO_2_ levels above the limit of 1000 ppm were observed in spring campaign, in the weeks close to the end of the COVID-19 quarantine, likely associated with the increased time that the occupants spent at home.

## 1. Introduction

Nowadays, there is an increasing interest in investigating and monitoring air quality of the indoor environments, since in the world becoming increasingly urbanized, urban residents typically spend 80–90% of their time in various indoor environments, such as homes, schools, offices, and restaurants. Thus, having good indoor air quality (IAQ) in those spaces appears to be essential, since exposure to components of indoor air has a direct influence on human health [1,2,3,4,5,6]. In fact, exposure to high concentrations of indoor air pollutants has been found to cause both acute and chronic health effects, including respiratory and cardiovascular illness, allergic symptoms, cancers, and premature mortality [1,2,4]. Therefore, it is important to characterize IAQ and understand which pollution sources, housing characteristics, and occupancy behaviors have the largest impact on human exposure to pollutants present in the home environment [4,6,7,8].

Major indoor pollutants include CO, CO_2_, volatile organic compounds (VOCs), PM_2.5_, and PM_10_, that are produced by a combination of numerous sources. They include indoor sources—i.e., furniture, utility, and building materials—, the presence of occupants and their activities, in particular, burning gas and other fossil fuels for cooking and heating, tobacco smoking, cleaning with detergents and personal care products, burning candles or incense [4,7,8,9]. In addition, a significant contribution to IAQ may derive from outdoor pollutants entering inside the rooms through ventilation systems, the physical opening of doors and windows and building envelope cracks [10,11,12]. Overall, the most effective strategies to prevent indoor pollution consist of control of emission sources combined with proper ventilation rates to dilute contaminants [13,14,15]. While the relationship between ventilation rates and indoor contaminant levels can be complex due to transient effects, studies have shown that increased ventilation rates are associated with reductions in the prevalence of sick building syndrome symptoms. More recently, there has been a focus on low-energy and even net-zero energy buildings, leading to building construction or renovation with increased energy efficiency by reducing heating and cooling loads through improving the thermal integrity of the envelope, increasing the efficiency of heating and cooling equipment and reducing system energy use through effective control approaches [15,16,17,18]. In this context, the building air tightness and thermal insulation, although the key factors for thermal performance and energy saving, may negatively affect the levels of gaseous and particulate indoor air pollutants, with consequent heath impacts of inhabitants [19,20,21]. Thus, given the importance of evaluating the impacts of sustainable, low-energy technologies to achieve good IAQ in high-performance buildings, there is the need to measure and continuously in situ monitoring IAQ in order to optimize ventilation systems for energy reductions.

In this context, low-cost air quality sensors are useful tools to economically monitor air quality in near real time to collect high-density temporal and spatial data in a broader range of households [22,23,24,25,26,27]. Although these monitors have analytical limitations compared with scientific instruments—with unfeasible cost and complexity for large-scale projects—they can guarantee the levels of performance suited to IAQ monitoring, in terms of precision, accuracy, sensitivity (detection limits), and time resolution. They are easy-to-use and convenient devices, provided with user-friendly interface, low maintenance requirement, low-power consumption, and battery-operation. In addition, they offer advantages of miniaturization of size, noisiness, and intrusiveness, when measuring in occupied spaces. Sensors can communicate data via Bluetooth or Wi-Fi to a remote platform (e.g., smartphone or a PC) to view, analyze, and interpret the data, so that users are directly informed on the presence of pollutants in the air they are breathing.

Within this frame, the purpose of the present work was to experimentally study the indoor air quality in terms of PM_2.5_, VOCs, and CO_2_ in two dwellings in several days covering winter, spring, and summer, in order to include different outdoor concentration levels. Attention was focused on outdoor PM_2.5_ pollution, that peaked over the EU level during winter and dropped in spring, just after the lockdown period imposed by the Covid-19 pandemic [28,29,30].

## 2. Materials and Methods

### 2.1. Study Area Description

The current research was performed in two homes in two sites in Emilia–Romagna region, located in the Eastern Po Valley (Northern Italy): one is Ferrara (~130,000 inhabitants) and the other is Medolla (~6000 inhabitants) located in the plain 50 km far from Ferrara.

Two weeks monitoring campaigns were carried out in the different seasons in 2020: in winter from 28 January–15 February, in spring from 23 May–6 June, and in summer, from 29 June–13 July.

### 2.2. Apartment Characteristics

The two investigated dwellings, both located at the second floor in a multi-storey residential building, used different energy efficiency. Flat 1 in Medolla is a contemporary energy efficient house built after the earthquake in 2012, using innovative design strategies to reduce energy consumption and make home more sustainable. Such strategies are mainly based on increasing airtightness of building envelopes, and the use of new controlled mechanical ventilation technologies and new building materials to reduce release into the indoor environment [17,22,31]. Flat 2 in Ferrara is a relatively old apartment built in the 70s, equipped with standard devices, i.e., natural ventilation, gas cookers. A family of five components with 4 pets live in flat 1, while 3 persons live in flat 2. No smokers are present in both dwellings. Features and occupation levels of the investigated flats are summarized in Table 1.

### 2.3. IAQ Monitoring

Indoor temperature, relative humidity (RH), PM_2.5_, VOCs, and CO_2_ concentrations were in situ measured using Foobot sensors purchased from Foobot (AirBoxLab, Luxembourg). Foobot is a passive device, which relies on natural convection to passively move air to the sensor.

To measure PM_2.5_ concentrations, the sensor utilizes a Sharp PM sensor, based on light scattering technology, that uses an infrared light emitting diode optical sensor and a photodiode detector to detect light scattered by particles passing its detection chamber. The light scattering data are then converted into PM_2.5_ mass concentration using a calibration curve [32]. Particles with an aerodynamic diameter between 0.3 and 2.5 μm can be measured in the concentration range of 0–1300 μg m^−3^, with a precision of ±4 μg m^−3^ [22]. Studies in laboratory and field conditions showed that the Foobot device provides good accuracy, compared with scientific instruments [22,23,24,27]. Foobot also measures total volatile organic compounds (VOCs) through a metal-oxide semiconductor (MOX) sensor in a concentration range of 125–1000 ppb, with a precision of ±1.0 ppb. The Foobot lacks a CO_2_ sensor and an algorithm converts total VOCs concentration into a CO_2_ equivalent using an equation developed by the producer [32] (range 400–6000 ppm, with precision ±1.0 ppm). The sensor does not report absolute values for any particular gas, but indicates the relative change in levels of a wide range of VOCs. This characteristic is suitable for IAQ monitoring, where a principal objective is to investigate the relative concentration of pollutants and their trends. The device is also equipped with a temperature and relative humidity sensor to measure T (range: −40 °C ± 125 °C, accuracy ± 0.4 °C) and RH (range: 0–100%, accuracy ± 4% RH) [22].

The Foobot device mechanism is based on a microprocessor that collects the electrical outputs from the sensors and converts them into data, which are then transmitted wirelessly to a remote server, where an algorithm is applied to derive the measured concentrations. The acquired data can be downloaded by the operator via Wi-Fi to a smartphone or a PC for viewing and analysis [22]. Recent papers, comparing the most common market low-cost sensors in indoor residential measurements report that Foobot is one of the most suitable devices to provide adequately reliable data for IAQ in situ monitoring of indoor environments [22,23,24,25,26,27].

Before each monitoring campaign, the 4 used Foobot devices were inter-calibrated by performing simultaneous measurements in the same laboratory for 3 consecutive days: the obtained values showed SD variation within the manufacturer’s expected range (data reported in Supplementary Materials Table S1) [22,32].

Measurements were performed simultaneously in the two dwellings during the three campaigns. Two measuring stations were selected in each flat, one in the kitchen and the other in the main bedroom, in order to likely single out the specific contribution of the cooking activity from the general occupant presence. Two weeks monitoring campaigns were carried out in the different seasons in 2020: in winter from 28 January–15 February, in spring from 23 May–6 June, and in summer from 29 June–13 July.

### 2.4. Meteorological and Atmoshere Conditions Characteristics

The outdoor temperature and PM_2.5_ concentrations were retrieved from the database of the Regional Environment Protection Agency of Emilia Romagna (ARPAE), that daily monitored air quality parameters at network air monitoring stations located in Ferrara and Medolla. PM_2.5_ mass concentrations were measured following the EN 12341:2014 standard gravimetric measurement method [33]. Daily PM_2.5_ samples were collected with low volume sampler (Skypost PM, TCR TECORA Instruments, Corsico, Milan, Italy) operating at the standard flow-rate of 38.3 L min^−1^ to collect an air volume of 55 m^3^ per day.

## 3. Results

### 3.1. Outdoor Parameters

The mean outdoor temperature, relative humidity, and PM_2.5_ concentration values were calculated for each monitoring period (±standard deviation) from the daily measured data and reported in Table 2.

The outdoor temperature followed the typical season trend in Northern Italy, with cold winter, even if exceptionally mild in this study with mean value ≈12 °C, increasing up to ≈30 °C in the hot summer.

The measured outdoor PM_2.5_ levels showed large differences among the seasons, as depicted by the time series reported in detail in the insets of Supplementary Materials Figure S1a–c. The highest values were observed in winter, with the widest variation from 27 to 72 µg m^−3^, so levels largely exceeded the EU limit of 25 µg m^−3^ [34]. Lower values were measured in spring/summer, ranging from 7 µg to 15 µg m^−3^. Such values with a strong seasonality and pollution winter episodes have been frequently observed in the study region, as well as in the whole Po Valley. This trend has been related to the seasonal cycle of Planetary Boundary Layer dynamics, with stable conditions in winter, that promote the pollutants accumulation in the first hundred meters of the atmosphere and thus favor atmospheric reactions generating relevant concentrations of secondary PM [35,36,37,38,39]. In addition, anthropogenic emission sources strengthen in fall/winter, with the increasing contribution of wood combustion for domestic heating, in addition to traffic and industry dominating sources. As a consequence of the stable atmospheric conditions, in general PM_2.5_ concentrations showed similar values at both urban and rural sites (non-significant difference at *p* < 0.05), mostly in the winter (inset of Supplementary Materials Figure S1a).

The PM_2.5_ values (7–9 µg m^−3^) measured in the May-June campaign were exceptionally low and homogeneous (inset of Supplementary Materials Figure S1b) in comparison with the levels commonly measured in spring/summer in the region [35,36,37,38,39]. This may be associated with the proximity of the study period to the end of the lockdown issued by the Italian government to prevent the spread of the COVID-19 infection. It imposed shut down of all non-essential factories and economic activities, closure of non-essential shops, schools and universities, banning of any gathering, household confinement of all people, with exception of key workers. As a consequence of the substantial reduction of the anthropogenic emissions, air quality has been found improved in the whole Po Valley, with significant reductions, up to 40% for NO_X_ and 20% for PM_10_, by nearly 50% reduction of PM concentrations [28,30].

### 3.2. Indoor AIQ Parameters

For each monitoring day of the three campaigns, indoor temperature, relative humidity, as well as PM_2.5_, VOCs, and CO_2_ concentrations were simultaneously monitored in the kitchen and bedroom of the two dwellings [22,23,24,25,39]. From the 24 h measured data, a whole daily value was computed, as well as separate values from daytime (from 9:00 a.m. to 9:00 p.m.) and night (from 9:00 p.m. to 9:00 a.m.) hours. From these data, the mean values were calculated for each monitoring period (±standard deviation) and reported in Table 2 (whole day) and Table 3 (daytime and night value, separately).

#### 3.2.1. Indoor Temperature

Overall, the indoor temperatures measured in the investigated rooms showed a small increase following the outdoor temperature, i.e., 20.7 ± 0.7 °C in winter, 24.2 ± 1.2 °C in spring, and 27.3 ± 1.8 °C in summer (Table 2). All measured values fall within the range recommended for indoor environment by the American National Standard Institute (ASHRAE), specifically 20–23 °C in winter and 23–27 °C in summer [40]. In order to guarantee the thermal comfort inside the rooms, in winter (T_out_ ≈ 12 °C) a heating system was operating, and windows and doors were kept close for most of the time. Similarly, in summer (T_out_ ≈ 30 °C) air conditioning system was operating with windows and doors mostly closed.

To highlight the impact of this behavior, the correlation between indoor and outdoor temperatures was investigated by conducting Pearson analysis. We graded the strength of the correlation in terms of the Pearson correlation coefficient (r) as moderate: 0.5 < r < 0.7, and strong: r ≥ 0.7 (Table 4). In winter and summer, the lack of significant correlation (r < 0.7) confirmed a limited air exchange from outside in all the investigated rooms. The same was also observed in spring in flat 1, where a forced air recirculating was operating. Otherwise, in the traditional dwelling 2, a moderate (r = 0.7) to strong (r = 0.8) correlation was found between T_in_ and T_out_ (Table 4), as windows were mainly open in the mild spring (T_out_ ≈ 25 °C), allowing a strong air exchange.

Overall, the relative humidity measured in the investigated rooms showed nearly constant values in the three monitoring periods, with mean values ranging from 46.3 ± 2.9 in Bed 2 in summer to 52.2 ± 4.1 in Bed 2 in spring (Table 2). All the data are inside the range of 45–55% recommended by ASHRAE for health and comfort [40], despite the humid climate of the Po Valley, mainly in winter with outdoor RH% up 81.6 ± 11.8 (Table 2) [30,35,39].

#### 3.2.2. Indoor PM_2.5_

The mean indoor PM_2.5_ concentrations were about 15 μg m^−3^ in winter, then they dropped to values close to 10 μg m^−3^ in spring and increased again in summer with values largely scattered about 13 μg m^−3^ (Table 2). The time series of the indoor PM_2.5_ levels are reported in detail in Supplementary Materials Figure S1a–c for the four investigated rooms during each campaign. Overall, the measured values are inside the wide range reported in literature studies in Europe [17,22,39,41,42,43].

**Figure 1:**
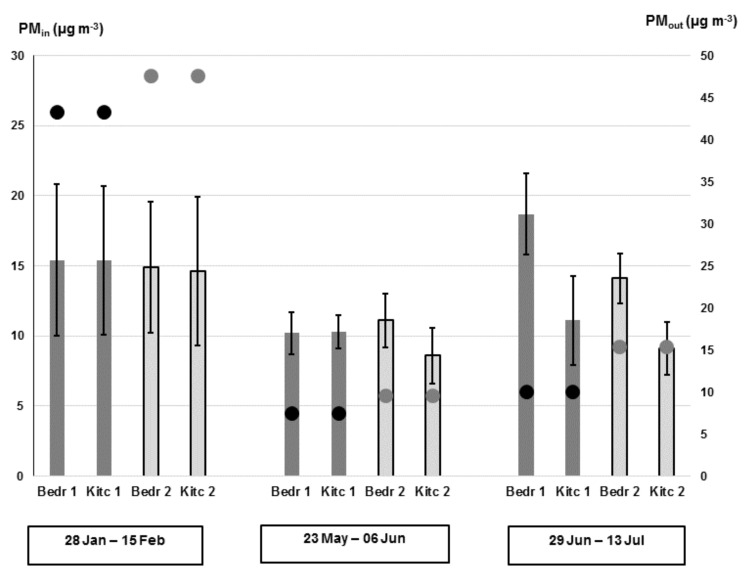
Mean PM_2.5_ concentrations measured in the three monitoring campaigns (indicated in the bottom insets). Histograms (left scale): indoor PM_2.5_ values measured in each room, kitchen and bedroom; points (right scale): outdoor PM_2.5_ values. Error bars represent one standard deviation of the mean. Dark grey histograms and black points represent flat 1 in rural site, light grey histograms and dark grey points represents flat 2 in urban site.

The mean indoor PM_2.5_ values measured in each room during the three campaigns are reported in Figure 1, where also mean outdoor PM_2.5_ data are shown for comparison.

In winter, we observe that the indoor PM_2.5_ values are largely scattered around the mean value of 15 μg m^−3^, following a daily trend that strongly resembles that of indoor PM_2.5_ (Supplementary Materials Figure S1a). Overall, indoor levels were lower than outdoors, so that the comfort conditions of 25 μg m^−3^ imposed by the WHO guidelines [44] can be satisfied, even when the outdoor PM_2.5_ levels peaked up to 45.0 μg m^−3^ (Table 2). It can be noted that the measured indoor PM_2.5_ values were nearly constant in the four monitored environments, with not significant difference (*p* < 0.05) between dwellings and rooms. This similarity may suggest that indoor PM_2.5_ mass concentration was mostly impacted by entrance from outdoor, compared with emission from indoor sources. This hypothesis was verified by investigating the correlation between indoor and outdoor PM_2.5_ concentrations by Pearson’s regression analysis. The obtained coefficients (in the range 0.73–0.86) showed strong correlation for all the rooms, so confirming the dominant role of the outdoor PM_2.5_ (Table 4). This is consistent with previous papers, that showed that a great portion of indoor particulates originates from the outdoor air that enters the indoor environment via the natural ventilation when windows are open, mechanical ventilation, that forces the air recirculation, and penetration through windows frames and/or cracks in building envelopes [5,17,18,20,26]. This last process can be supposed as the dominating mechanism in the study period, as the air exchange was limited through the windows, that were kept closed for most of the time, as confirmed by investigation on T_in_ data. The dominant impact of entrance from outdoor may be motivated by the high outdoor PM_2.5_ levels, as commonly found in industrial sites or urban areas, generating a high exposure to airborne contaminants inside homes [9,10,11,41,43,45,46,47]. In additions, a deep insight into the data separated between day (from 9:00 a.m. to 9:00 p.m.) and night (from 9:00 p.m. to 9:00 a.m.) hours show that in the kitchen of both apartments the PM_2.5_ concentration significantly increased during the daytime compared with the night (Table 3). This trend suggests the concomitant contribution of internal sources operating in these rooms, mainly related with cooking and occupant activity [5,12,14,21,27,43].

Overall, the role played by infiltration from high outdoor PM_2.5_ levels, can be investigated by computing the PM_2.5_ I/O ratio, as a commonly used parameter to represent the relative strength of indoor air pollutant concentration with respect to the immediate outdoor environment (Table 2). In winter, the measured values ranged from 0.33 ± 0.08 to 0.37 ± 0.10, that are lower than the reference value about 0.7 commonly found in dwellings without any indoor sources [5,39]. The values largely lower than 1 indicate that the penetration through building physical barriers can largely remove particles, and thus strongly reduce the PM exposition experienced by persons inside the rooms in comparison with outdoors. This is very relevant from the toxicological point of view, mainly when outdoor PM_2.5_ concentration is too high [11,45,46,47]. It can be noted that the strong similarity among the values in the four monitored environments confirmed the poor contribution of PM_2.5_ emission from indoor sources.

A deep insight into the measured data in the spring and summer campaigns (daily trends reported in Supplementary Materials Figure S1b,c for each investigated room) showed significant (*p* < 0.05) statistical variations between the two dwellings, with higher values in flat 1 (Table 2). Such differences were further magnified by discriminating between daytime and night periods, with the lowest value in the kitchen of flat 2 during the night (7.2 ± 1.8 µg m^−3^) (Table 3). This pattern may suggest the concomitant contribution of internal sources operating in these rooms, other than income from outdoor. The major contribution of indoor PM_2.5_ has been found related to domestic activities like cooking, cleaning, and the constant presence of occupants inside the dwellings, that release particles, mainly skin fragments, hairs, fibers from clothes, dandruff, animal hairs, and resuspend particles previously deposited on indoor surfaces [10,11,12,41,43]. This motivates the higher values found in home 1 with more occupants (5 persons and 3 pets) compared with home 2 (3 persons), mainly in the kitchen. Moreover, the spring data highlighted that the advanced thermo/energy regulation system operating in flat 1 made PM_2.5_ concentrations nearly constant inside the rooms during the whole day (mean values close to 10 µg m^−3^, with not significant day/night and kitchen/bedroom variations), in contrast with the simple traditional system present in flat 2 (values ranging from 7.2 ± 1.8 µg m^−3^ to 11.2 ± 1.86 µg m^−3^, Table 3). Even if some outdoor infiltration was operating, its impact is expected poor, as outdoor PM_2.5_ concentration was low in the investigated periods (insets in Supplementary Materials Figure S1b,c), in particular in the spring campaign. This may be related to the reduced emissions from vehicular traffic and activity of non-essential factories, that were still stopped, as they restarted only gradually after the pandemic lockdown [28,30]. The dominant contribution of PM_2.5_ from indoor release is confirmed by the lack of significant correlation between indoor and outdoor PM_2.5_ concentrations (Table 4) for all the investigated rooms. Otherwise, in summer, the Pearson’s regression analysis showed moderate correlation between indoor and outdoor PM_2.5_ values for rooms of flat 2 (r in the range 0.68–0.70) (Table 4). This can be explained by the frequent opening of windows in the hot period, consistent with the limited operation of the traditional cooling system.

For spring and summer data, the PM_2.5_ indoor-to-outdoor concentration ratio was mostly ≥1 for all the investigated rooms, in contrast with the low winter value (Table 2). In general, the computed values showed significant (*p* < 0.05) variation between dwellings and between kitchen and bedroom in the same flat, with values ranging from 0.74 ± 0.23 in the kitchen of flat 1 to 1.59 ± 0.52 in the bedroom of flat 2. Both these results confirm the combined contribution of PM_2.5_ indoor domestic sources, other than outdoor infiltration.

#### 3.2.3. VOCs and CO_2_

**Figure 2:**
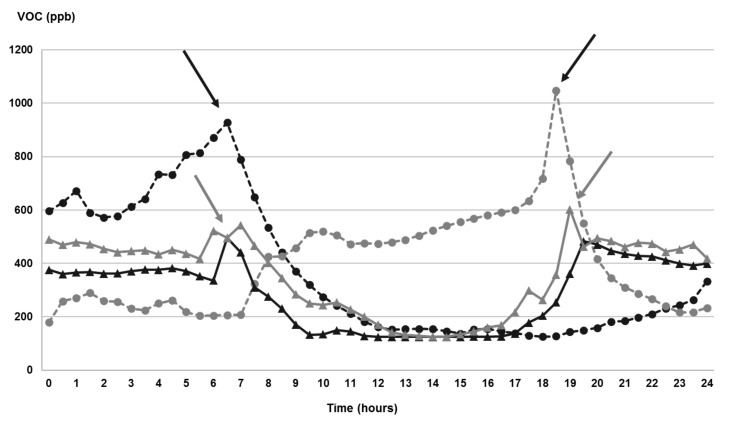
Daily (10 February 2020) evolution of VOCs concentration in each investigated room. Points (dashed lines) represent kitchens and triangles (full lines) bedrooms; black symbols and lines correspond to flat 1; light grey symbols and lines correspond to flat 2. Arrows indicate the occupant activities: black arrows indicate occupants’ cooking activities, while light grey ones indicate go to bed/wake up.

Overall, the measured concentrations of volatile organic compounds showed quite homogenous values ranging from 260 ± 52 ppb to 317 ± 91 ppb in winter, between 343 ± 60 ppb and 584 ± 92 in May and from 131 ± 52 ppb to 491 ± 105 in summer (Table 2). The time series of the measured indoor VOCs levels are reported in detail in Supplementary Materials Figure S2a–c for the four investigated rooms during each campaign. The measured data are close to the upper end of the range of values reported in other European sites [17,18,39]. The presence of VOCs (such as formaldehyde, terpenes) has been found mainly associated with emission from building materials including carpet, plywood, paint, and also occupants’ activity involving the use of chemicals in daily housework and personal care and also with specific household activities, such as cooking or leisure [48]. Moreover, VOCs have been found to increase with the occupant density, being associated with emissions with breath and metabolic processes, as well as with movement of occupants inside internal spaces [7,21]. An example of the relationship of VOCs concentration with human activity is clearly shown in Figure 2, that reports the evolution of VOCs level during a whole day (10 February 2020) in the four investigated rooms.

Overall, we can see similar trends in the bedrooms of both flats, with two peaks close to 7 a.m. and 7–8 p.m., that may be related to the occupants’ morning rise and go to bed at night. Otherwise, we observe opposite trends in the two kitchens, with a peak at 7 a.m., in flat 1, likely related to the occupants’ breakfast, and a peak at 7 p.m. in kitchen 2, probably associated with dinner. In general, significantly higher values were mostly measured during the daytime hours, compared with night period in most of the investigated rooms (Table 3). Among the investigated periods, the highest values were measured during the spring campaign, with mean values 584 ± 92 ppb and 444 ± 96 ppb in flat 1 and 2, respectively. This may be generated by the increased time that occupants spent at home, due to imposition of teleworking and confinement, since most of the work activities outside home restarted only gradually at the end of the pandemic lockdown. In addition, the domestic use of cleaning and disinfection products was enhanced for protection against the COVID-19 virus [28,29,30]. A detailed inspection of the daily trends in the three campaigns (Supplementary Materials Figure S2a–c) shows larger differences between the flats in spring and summer, compared with winter. In general, higher values were observed in flat 1 than in flat 2, that may be associated with the higher occupancy level of human and pets (Table 2 and Table 3). The specific contribution of VOCs sources compared with indoor PM_2.5_ origins was investigated by relating indoor concentration of VOCs with that of PM_2.5_ for each monitored room. In general, no significant correlation was found, suggesting that these pollutants are generated from a different combination of independent emission sources and/or infiltration from outdoor sources in the three investigated periods.

Carbon dioxide concentration follows the same VOCs pattern, since CO_2_ equivalent was estimated from the experimentally measured VOCs values using an algorithm conversion [32]. CO_2_ values ranged between 940 ± 190 ppm and 1147 ± 331 ppm in winter, from 1240 ± 219 ppm to 2116 ± 517 ppm in spring, and from 470 ± 19 to 1783 ± 383 ppm in summer (Table 2). Nearly all the measured CO_2_ levels were above the limit of 1000 ppm imposed by ASHRAE [40]. This suggests that in the investigated dwellings the ventilation systems were not able of ensuring adequate air-exchange, with potential risk of air-quality related issues. It can be noted that such a problematic situation was found in both dwellings, even if an advanced mechanical ventilation system was operating in the flat 1, differently from the naturally ventilated flat 2. This may be likely associated with the larger number of occupants, with consequent higher emissions through breathing and metabolic processes, but may be also the result of faults in the system, occupant interference, poor installation, and/or lack of maintenance [13,17,19,31,47].

## 4. Conclusions

The indoor air quality of two dwellings was assessed during three experimental campaigns performed in different seasons, characterized by varying outdoor atmospheric characteristics, mainly PM_2.5_ concentrations. The use of low-cost sensors, able to continuously monitor IAQ parameters, has been found suitable for in situ home monitoring, taking the advantage of simplicity, speed, and data availability. The acquired data can be immediately downloaded and displayed on smartphones by the occupants to develop awareness on the air quality inside the homes where they live.

High outdoor PM_2.5_ pollution was found to negatively influence IAQ, since it was mainly responsible of indoor PM_2.5_, as a consequence of particle penetration inside the rooms through windows and/or cracks in building envelopes. The finding of elevated CO_2_ levels under typical occupancy conditions suggests inadequate ventilation in both the naturally ventilated and mechanically ventilated dwellings.

As this study included only one new energy efficient dwelling compared with one conventional apartment, any generalization of the results is not possible to give information on the still open question of impact of low-energy strategies on IAQ. Thus, further research is required to highlight the best strategies capable of ensuring adequate ventilation, including the design, construction, operation, and maintenance of ventilation systems. Particular attention must be paid on this point, in the possible prospect of other total or partial quarantine scenarios imposed by sanitary emergency (COVID-19 next waves), that may further aggravate the situation associated with the increased time that people spend at home.

## Figures and Tables

**Table 1:** Features of dwellings monitored in the study.

Feature	Flat 1	Flat 2
Location	Rural (Medolla)	Urban (Ferrara)
Year of construction	2012	1970
N° occupants	5	3
N° pets	4	-
Ventilation system	Mechanically controlled windows Forced air recirculating	Natural ventilation
Heating system	Underfloor heating	Radiators
Cooling system	Heat pump (whole house unit)	-
Air conditioning	Underfloor air conditioning	Split units in rooms
Cookers	Induction	Gas

**Table 2:** Indoor and outdoor daily parameters (24 h) measured during each monitoring campaign in each investigated room (campaign mean ± standard deviation values).

Period	T_out_ (°C)	RH%_out_	Outdoor PM_2.5_ (µg m^−3^)	Room	T_in_ (°C)	RH%_in_	Indoor PM_2.5_ (µg m^−3^)	PM_2.5_ I/O Ratio	VOCs (ppb)	CO_2_ (ppm)
28 January–15 February	12.0 ± 2.5	81.6 ± 11.8	43.3 ± 16.4	Bed 1	20.2 ± 0.4	48.6 ± 2.3	15.4 ± 5.4	0.35 ± 0.12	306 ± 47	1106 ± 170
				Kitc 1	19.9 ± 0.2	48.4 ± 2.3	15.4 ± 5.3	0.33 ± 0.08	302 ± 54	1094 ± 196
	11.7 ± 2.3	75.8 ± 15.8	47.6 ± 17.1	Bed 2	21.2 ± 0.3	48.5 ± 2.3	14.9 ± 4.7	0.37 ± 0.10	260 ± 52	940 ± 190
				Kitc 2	21.4 ± 0.2	48.6 ± 2.3	14.6 ± 5.3	0.34 ± 0.08	317 ± 91	1147 ± 331
23 May–6 June	25.7 ± 3.5	63.5 ± 11.3	7.4 ± 1.6	Bed 1	22.8 ± 0.3	52.2 ± 4.1	10.2 ± 1.5	1.12 ± 0.38	373 ± 66	1349 ± 238
				Kitc 1	23.4 ± 0.4	52.0 ± 4.0	10.3 ± 1.2 *^#^*	1.13 ± 0.33	584 ± 92 ^#^	2116 ± 517 ^#^
	24.1 ± 3.2	54.3 ± 12.5	9.6 ± 2.1	Bed 2	25.5 ± 0.6	52.1 ± 4.0	11.1 ± 1.9	1.59 ± 0.52	444 ± 96	1610 ± 347
				Kitc 2	25.0 ± 0.6	52.0 ± 3.9	8.6 ± 2.0	1.22 ± 0.43	343 ± 60	1240 ± 219
29 June–13 July	30.8 ± 2.8	63.4 ± 6.2	10.0 ± 2.4	Kitc 1	29.7 ± 1.0	46.9 ± 3.4	18.7 ± 2.9 ^#^	1.21 ± 0.26	271 ± 55 ^#^	954 ± 228 ^#^
				Kitc 1	24.7 ± 0.3	47.0 ± 3.3	11.1 ± 2.2 ^#^	0.74 ± 0.23	491 ± 85 ^#^	1783 ± 383 ^#^
	30.0 ± 2.7	63.4 ± 6.2	15.4 ± 3.9	Bed 2	27.5 ± 0.4	46.3 ± 2.9	14.1 ± 1.8	1.47 ± 0.39	131 ± 52	470 ± 19
				Kitc 2	27.2 ± 0.5	46.4 ± 3.0	9.1 ± 1.9	0.94 ± 0.26	361 ± 79	1284 ± 411

^#^ significant (*p* < 0.05) difference of PM_2.5_ and volatile organic compounds (VOCs) concentrations between flat 1 and flat 2.

**Table 3:** Indoor PM_2.5_ and VOCs concentrations measured during each monitoring campaign in each investigated room discriminated between daytime (from 9:00 a.m. to 9:00 p.m.) and night (from 9:00 p.m. to 9:00 a.m.) hours.

Period	Room	Daytime PM_2.5_ (µg m^−3^)	Night PM_2.5_ (µg m^−3^)	Daytime VOCs (ppb)	Night VOCs (ppb)
28 January–15 February	Bed 1	17.1 ± 7.4	13.1 ± 5.4	437 ± 92 *	156 ± 15
	Kitc 1	19.1 ± 8.0 *	10.4 ± 5.3	372 ± 89 *	231 ± 32
	Bed 2	15.8 ± 6.3	13.1 ± 4.7	314 ± 83 *	219 ± 39
	Kitc 2	17.6 ± 6.5 *	11.1 ± 4.7	355 ± 93 *	287 ± 75
23 May–6 June	Bed 1	10.6 ± 17	9.6 ± 1.8	561 ± 139 *	294 ± 52
	Kitc 1	10.5 ± 1.6	10.0 ± 1.61 ^#^	841 ± 185 *	479 ± 136
	Bed 2	10.9 ± 2.7	11.2 ± 1.61	576 ± 140 *	384 ± 101
	Kitc 2	9.9 ± 3.1 *	7.2 ± 1.8	394 ± 82	317 ± 60
29 June–13 July	Bed 1	19.0 ± 5.1	18.3 ± 4.7 ^#^	278 ± 68 ^#^	249 ± 46 ^#^
	Kitc 1	11.6 ± 5.7	10.4 ± 1.3 ^#^	630 ± 178 *^#^	348 ± 72
	Bed2	14.4 ± 1.7	13.7 ± 1.8	128 ± 3	133 ± 8
	Kitc 2	9.4 ± 3.5	8.9 ± 1.5	391 ± 113	317 ± 119

* significantly (*p* < 0.05) different values between daytime and night concentrations; ^#^ significant (*p* < 0.05) difference of PM_2.5_ and VOCs concentrations between flat 1 and flat 2.

**Table 4:** Pearson’s coefficients of correlation between the different indoor and outdoor parameters measured during each monitoring campaign in each investigated room: indoor vs. outdoor Temperature, indoor vs. outdoor PM_2.5_ concentrations. Bold values represent Pearson’s coefficient significant at *p* < 0.05 level.

Period	Room	Indoor vs. Outdoor Temperature	Indoor vs. Outdoor PM_2.5_
28 January–15 February	Bed 1	−0.057	0.734 **
	Kitc 1	−0.079	0.731 **
	Bed 2	0.307	0.842 **
	Kitc 2	0.244	0.789 **
23 May–6 June	Bed 1	−0.380	0.395
	Kitc 1	0.274	−0.035
	Bed 2	0.687 *	−0.293
	Kitc 2	0.802 **	−0.455
29 June–13 July	Bed 1	0.491	0.408
	Kitc 1	−0.063	0.299
	Bed 2	0.478	0.667 *
	Kitc 2	−0.271	0.656 *

* moderate correlation: 0.5 < r < 0.7; ** strong correlation: r ≥ 0.7.

## Data Availability

The data presented in this study are available on request from the corresponding author.

## Supplementary Materials

The following are available online at https://www.mdpi.com/article/10.3390/ijerph18084060/s1, Table S1: results of inter-calibration study of the 4 Foobot devices used in the study: mean and standard variation values of the IAQ parameters simultaneously measured in the same laboratory for 3 consecutive days before each monitoring campaign, Figure S1a–c: time series plots of indoor PM2.5 concentration during each monitoring campaign in the four investigated rooms; figure insets show times evolution of ooutdoor PM2.5 levels, Figure S2a–c: time series plots of VOCs concentration during each monitoring campaign in the four investigated rooms.

## References

[1] Health Effects Institute (2020). State of Global Air 2020.

[2] Shaddick G., Salter J.M., Peuch V.-H., Ruggeri G., Thomas M.L., Mudu P., Tarasova O., Baklanov A., Gumy S. (2021). Global Air Quality: An Inter-Disciplinary Approach to Exposure Assessment for Burden of Disease Analyses. Atmosphere.

[3] Settimo G., Manigrasso M., Avino P. (2020). Indoor air quality: A focus on the European legislation and state-of-the-art research in Italy. Atmosphere.

[4] Leung D.Y.C. (2015). Outdoor-indoor air pollution in urban environment: Challenges and opportunity. Front. Environ. Sci..

[5] Li Z., Wen Q., Zhang R. (2017). Sources, health effects and control strategies of indoor fine particulate matter (PM2.5): A review. Sci. Total Environ..

[6] Zar H.J., Ferkol T.W. (2014). The global burden of respiratory disease—Impact on child health. Pediatr. Pulmonol..

[7] Bari M.A., Kindzierski W.B., Wheeler A., Héroux M.È., Wallace L.A. (2015). Source apportionment of indoor and outdoor volatile organic compounds at homes in Edmonton, Canada. Build. Environ..

[8] Goel S.G., Somwanshi S., Mankar S., Srimuruganandam B., Gupta R. (2021). Characteristics of indoor air pollutants and estimation of their exposure dose. Air Qual. Atmos. Health.

[9] Yassin M.F., Al Thaqeb B.E.Y., Al-Mutiri E.A.E. (2012). Assessment of indoor PM_2.5_ in different residential environments. Atmos. Environ..

[10] Martins N.R., Carrilho da Graça G. (2018). Impact of PM_2.5_ in indoor urban environments: A review. Sustain. Cities Soc..

[11] Tofful L., Perrino C. (2015). Chemical composition of indoor and outdoor PM_2.5_ in three schools in the city of Rome. Atmosphere.

[12] Nadali A., Arfaeinia H., Asadgol Z., Fahiminia M. (2020). Indoor and outdoor concentration of PM_10_, PM_2.5_ and PM_1_ in residential building and evaluation of negative air ions (NAIs) in indoor PM removal. Environ. Pollut. Bioavailab..

[13] McGill G., Oyedele L.O., McAllister K. (2015). Case study investigation of indoor air quality in mechanically ventilated and naturally ventilated UK social housing. Int. J. Sustain. Built. Environ..

[14] Vardoulakis S., Giagloglou E., Steinle S., Davis A., Sleeuwenhoek A., Galea K.S., Dixon K., Crawford J.O. (2020). Indoor exposure to selected air pollutants in the home environment: A systematic review. Int. J. Environ. Res. Public Health.

[15] Sánka I., Földváry V. (2017). Indoor Air Quality of Residential Building Before and After Renovation. Slovak J. Civ. Eng..

[16] Földváry V., Bekö G., Langer S., Arrhenius K., Petráš D. (2017). Effect of energy renovation on indoor air quality in multifamily residential buildings in Slovakia. Build. Environ..

[17] Broderick Á., Byrne M., Armstrong S., Sheahan J., Coggins A.M. (2017). A pre and post evaluation of indoor air quality, ventilation, and thermal comfort in retrofitted co-operative social housing. Build. Environ..

[18] Schweizer C., Edwards R.D., Bayer-Oglesby L., Gauderman W.J., Ilacqua V., Jantunen M.J., Lai H.K., Nieuwenhuijsen M., Künzli N. (2007). Indoor time-microenvironment-activity patterns in seven regions of Europe. J. Expo. Sci. Environ. Epidemiol..

[19] Fernández-Agüera J., Domínguez-Amarillo S., Alonso C., Martín-Consuegra F. (2019). Thermal comfort and indoor air quality in low-income housing in Spain: The influence of airtightness and occupant behavior. Energy Build..

[20] Langer S., Bekö G., Bloom E., Widheden A., Ekberg L. (2015). Indoor air quality in passive and conventional new houses in Sweden. Build. Environ..

[21] Fernández-Agüera J., Dominguez-Amarillo S., Fornaciari M., Orlandi F. (2019). TVOCs and PM2.5 in naturally ventilated homes: Three case studies in a mild climate. Sustainability.

[22] Moreno-Rangel A., Sharpe T., Musau F., McGill G. (2018). Field evaluation of a low-cost indoor air quality monitor to quantify exposure to pollutants in residential environments. J. Sens. Sens. Syst..

[23] Sousan S., Koehler K., Hallett L., Peters T.M. (2017). Evaluation of consumer monitors to measure particulate matter. J. Aerosol. Sci..

[24] Liu X., Jayaratne R., Thai P., Kuhn T., Zing I., Christensen B., Lamont R., Dunbabin M. (2020). Low-cost sensors as an alternative for long-term air quality monitoring. Environ. Res..

[25] Chojer H., Branco P.T.B., Martins F., Alvim-Ferraz M.C.M., Sousa S.I.V. (2020). Development of low-cost indoor air quality monitoring devices: Recent advancements. Sci. Total Environ..

[26] Manibusan S., Mainelis G. (2020). Performance of four consumer-grade air pollution measurement devices in different residences. Aerosol. Air Qual. Res..

[27] Lowther S., Jones K.C., Wang X., Whyatt J.D., Wild O., Booker D. (2019). Particulate Matter Measurement Indoors: A Review of Metrics, Sensors, Needs, and Applications. Environ. Sci. Technol..

[28] Cameletti M. (2020). The Effect of Corona Virus Lockdown on Air Pollution: Evidence from the City of Brescia in Lombardia Region (Italy). Atmos. Environ..

[29] Saini J., Dutta M., Marques G. (2021). Indoor Air Quality Monitoring Systems and COVID-19. Emerg. Technol. Era COVID-19 Pandemic.

[30] PREPAIR-Po Regions Engaged to Policies of AIR. https://www.lifeprepair.eu/.

[31] Guyot G., Sherman M.H., Walker I.S. (2018). Smart ventilation energy and indoor air quality performance in residential buildings: A review. Energy Build..

[32] Indoor Air Quality Monitoring Systems and COVID-19” Is the Paper Title, iAQ-Core Indoor Air Quality Sensor Module. https://www.sciosense.com/wp-content/uploads/documents/iaQ-Core-Datasheet.pdf.

[33] (2014). EN:12341. https://www.gazzettaufficiale.it/atto/serie_generale/PubblicazioneGazzetta=2017-02-09.

[34] European Environmental Agency (2018). Air Quality in Europe—2018 Report No 12/2018. papers2://publication/uuid/1D25F41B-C673-4FDA-AB71-CC5A2AD97FDD.

[35] Decesari S., Allan J., Plass-Duelmer C., Williams B.J., Paglione M., Facchini M.C.C., O’Dowd C., Harrison R.M., Gietl J.K., Coe H. (2014). Measurements of the aerosol chemical composition and mixing state in the Po Valley using multiple spectroscopic techniques. Atmos. Chem. Phys..

[36] Bigi A., Ghermandi G., Harrison R.M. (2012). Analysis of the air pollution climate at a background site in the Po valley. J. Environ. Monit..

[37] Pietrogrande M.C., Bacco D., Ferrari S., Ricciardelli I., Scotto F., Trentini A., Visentin M. (2016). Characteristics and major sources of carbonaceous aerosols in PM_2.5_ in Emilia Romagna Region (Northern Italy) from four-year observations. Sci. Total Environ..

[38] Khan M.B., Masiol M., Formenton G., Di Gilio A., de Gennaro G., Agostinelli C., Pavoni B. (2016). Carbonaceous PM_2.5_ and secondary organic aerosol across the Veneto region (NE Italy). Sci. Total Environ..

[39] Zagatti E., Russo M., Pietrogrande M.C. (2020). On-Site Monitoring Indoor Air Quality in Schools: A Real-World Investigation to Engage High School Science Students. J. Chem. Educ..

[40] The American Society of Heating R and A-CE (ASHRAE) (2017). Thermal environmental conditions for human occupancy. ANSI/ASHRAE Stand-55.

[41] Santamouris M., Argiroudis K., Georgiou M., Pavlou K., Assimakopoulos M., Sfakianaki K. (2007). Indoor air quality in fifty residences in Athens. Int. J. Vent..

[42] Derbez M., Berthineau B., Cochet V., Lethrosne M., Pignon C., Riberon J., Kirchner S. (2014). Indoor air quality and comfort in seven newly built, energy-efficient houses in France. Build. Environ..

[43] Perrino C., Tofful L., Canepari S. (2016). Chemical characterization of indoor and outdoor fine particulate matter in an occupied apartment in Rome, Italy. Indoor Air.

[44] WHO Guidelines for Indoor Air Quality: Selected Pollutants. https://www.euro.who.int/__data/assets/pdf_file/0009/128169/e94535.pdf.

[45] Ruggieri S., Longo V., Perrino C., Canepari S., Drago G., L’Abbate L., Balzan M., Cuttitta G., Scaccianoce G., Minardi R. (2019). Indoor air quality in schools of a highly polluted south Mediterranean area. Indoor Air.

[46] Romagnoli P., Balducci C., Perilli M., Vichi F., Imperiali A., Cecinato A. (2016). Indoor air quality at life and work environments in Rome, Italy. Environ. Sci. Pollut. Res..

[47] Taylor J., Shrubsole C., Davies M., Biddulph P., Das P., Hamilton I., Vardoulakis S., Mavrogianni A., Jones B., Oikonomou E. (2014). The modifying effect of the building envelope on population exposure to PM_2.5_ from outdoor sources. Indoor Air.

[48] Sun X., He J., Yang X. (2017). Human breath as a source of VOCs in the built environment, Part II: Concentration levels, emission rates and factor analysis. Build. Environ..

