# Supplementary material for: Indoor Air Quality in Domestic Environments during Periods Close to Italian COVID-19 Lockdown
Source: Int J Environ Res Public Health. 2021 Apr 12;18(8):4060. doi: 10.3390/ijerph18084060 (PMC8070062; doi:10.3390/ijerph18084060)
Supplement: Supplementary file 1 [file ijerph-18-04060-s001.pdf]

International Journal of
Environmental Research and Public Health

MDPI

Indoor air quality in domestic environments during periods close to Italian COVID-19 lockdown

Maria Chiara Pietrogrande*, Lucia Casari, Giorgia Demaria, Mara Russo

Department of Chemical, Pharmaceutical and Agricultural Sciences, University of Ferrara,
Via Fossato di Mortara 17/19 - 44121 Ferrara, Italy;
mpc@unife (M.C.P); lucia.casari@unife.it (L.C.); dmrgrg@unife.it (G.D.); rssmra1@unife.it (M.R.)
* Correspondence: mpc@unife

Supplementary Materials

The Supplementary Materials contain the following elements:

Table S1: results of inter-calibration study of the 4 Foobot devices used in the study: mean and standard variation values of the IAQ parameters simultaneously measured in the same laboratory for 3 consecutive days before each monitoring campaign.

Figure S1. Time series plots of indoor $PM_{2.5}$ concentration during each monitoring campaign in the four investigated rooms. Points (dashed lines) represent kitchens and triangles (full lines) bedrooms; black symbols and lines correspond to flat 1; light grey symbols and lines correspond to flat 2. Figure insets show times evolution of ooutdoor $PM_{2.5}$ levels. S1a: winter campaign, 28 January to 15 February 2020; S1b: spring campaign, 23 May to 06 June 2020; S1c: summer campaign, 29 June to 13 July 2020.

Figure S2. Time series plots of VOCs concentration during each monitoring campaign in the four investigated rooms. Points (dashed lines) represent kitchens and triangles (full lines) bedrooms; black symbols and lines correspond to flat 1; light grey symbols and lines correspond to flat 2. S2a: winter campaign, 28 January to 15 February 2020; S2b: spring campaign, 23 May to 06 June 2020; S2c: summer campaign, 29 June to 13 July 2020.

Table S1: results of inter-calibration study of the 4 Foobot devices: mean and standard variation values of the IAQ parameters simultaneously measured in the same laboratory for 3 consecutive days before each monitoring campaign.

Period	T_{in} (C°)	$RH\%_{in}$	Indoor PM2.5 (μg m^{-3})	VOCs (ppb)	CO_2 (ppm)
25 to 27 Jan	20.2 ± 0.3	47.9 ± 1.9	15.2 ± 2.8	270 ± 1.0	978 ± 0.9
20 to 22 May	23.9 ± 0.3	50.2 ± 1.8	10.2 ± 1.9	285 ±0.9	1032 ± 0.95
25 to 27 Jun	25.7 ± 0.4	47.1 ± 1.4	11.7 ± 2.1	279 ± 1.0	1010 ± 1.0

a)

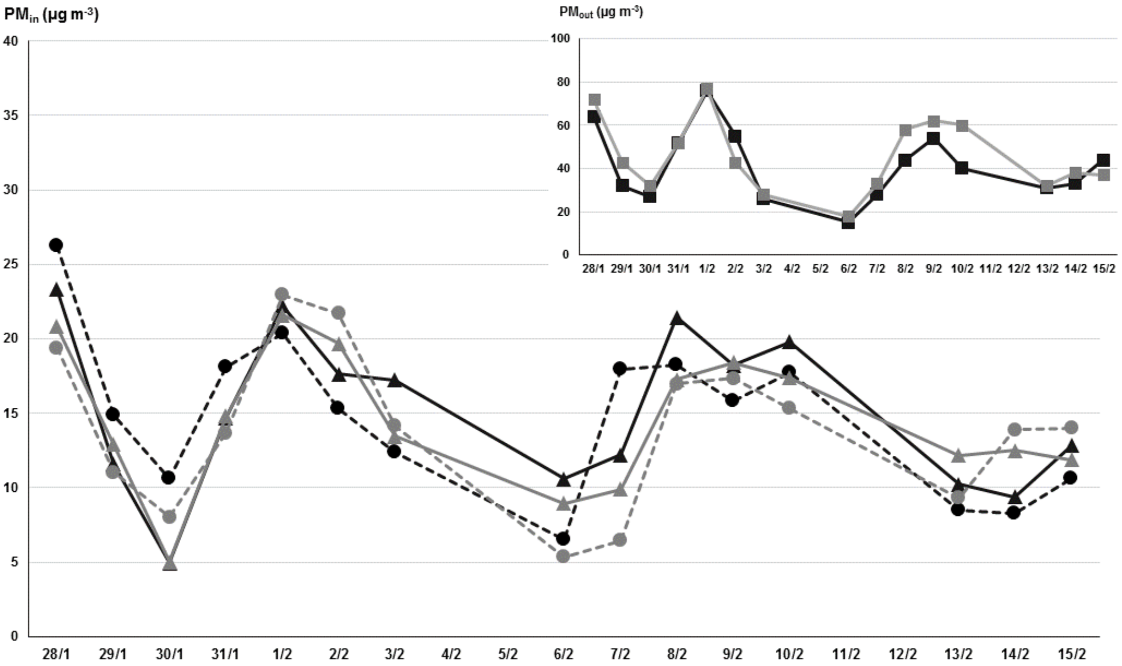

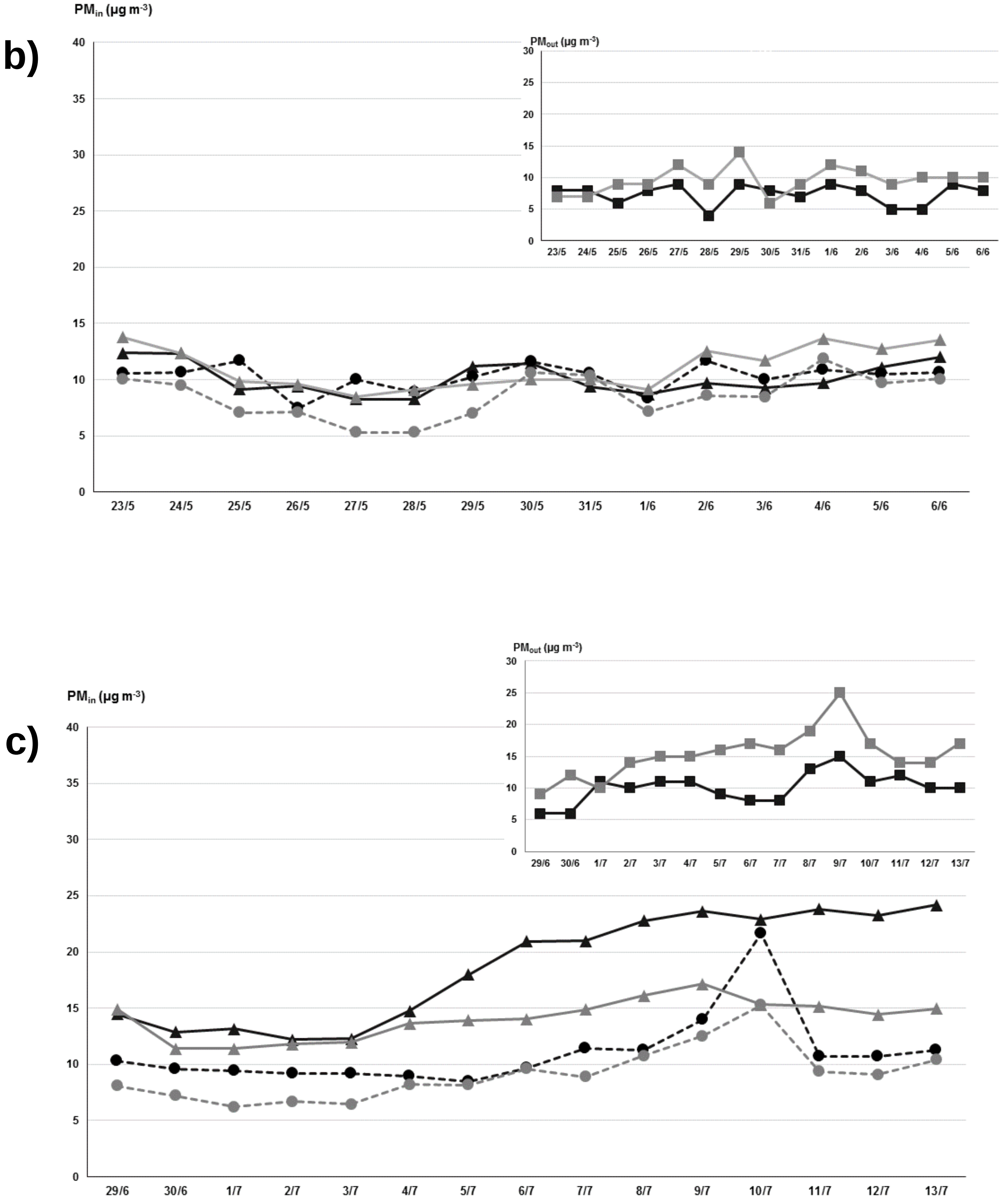

Figure S1. Time series plots of indoor $PM_{2.5}$ concentration during each monitoring campaign in the four investigated rooms. Points (dashed lines) represent kitchens and triangles (full lines) bedrooms; black symbols and lines correspond to flat 1; light grey symbols and lines correspond to flat 2. Figure insets show times evolution of ooutdoor $PM_{2.5}$ levels. S1a: winter campaign, 28 January to 15 February 2020; S1b: spring campaign, 23 May to 06 June 2020; S1c: summer campaign, 29 June to 13 July 2020.

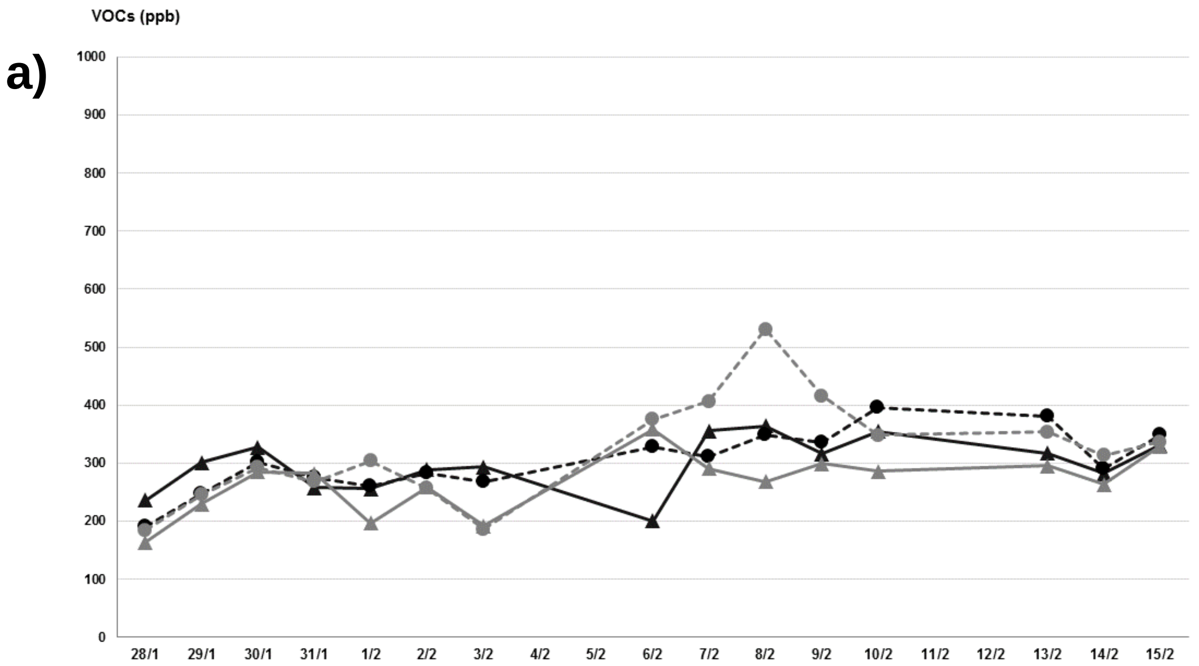
VOCs (ppb)
a)
1000
900
800
700
600
500
400
300
200
100
0
28/1
29/1
30/1
31/1
1/2
2/2
3/2
4/2
5/2
6/2
7/2
8/2
9/2
10/2
11/2
12/2
13/2
14/2
15/2

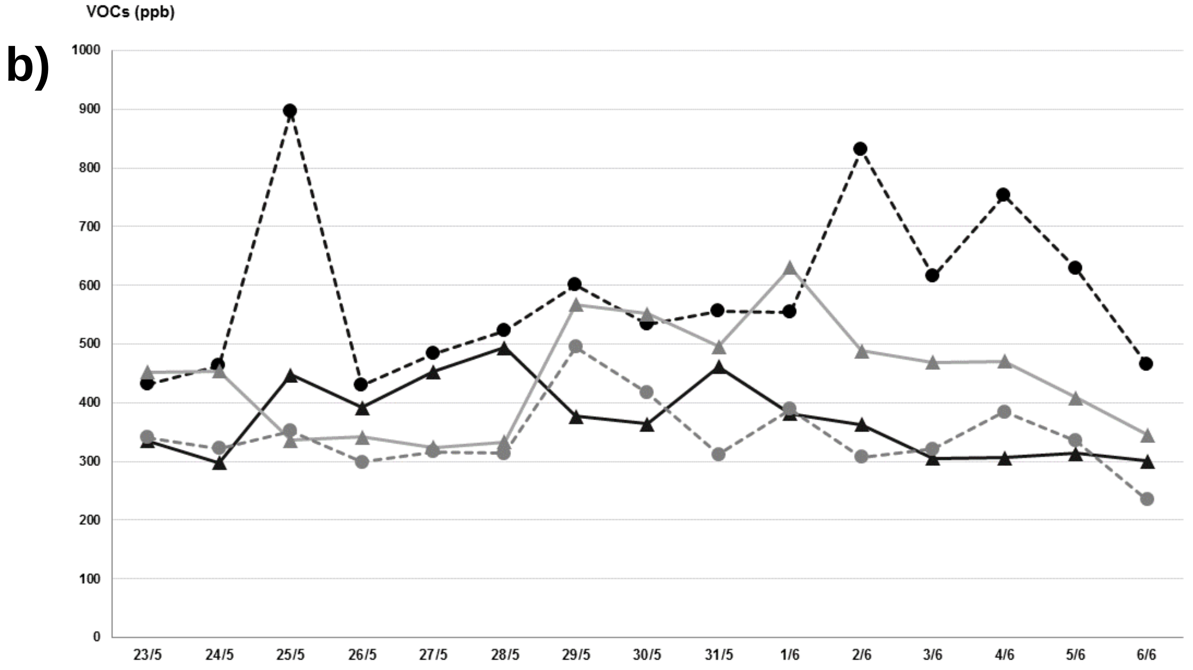
VOCs (ppb)
b)
1000
900
800
700
600
500
400
300
200
100
0
23/5
24/5
25/5
26/5
27/5
28/5
29/5
30/5
31/5
1/6
2/6
3/6
4/6
5/6
6/6

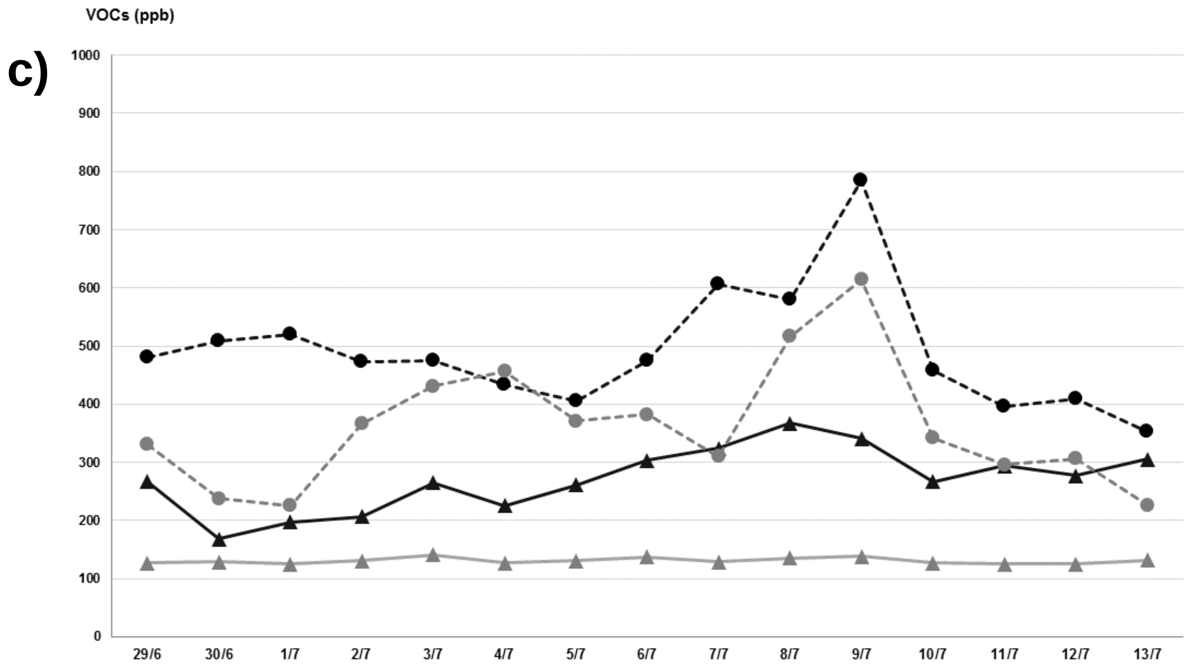

Figure S2. Time series plots of VOCs concentration during each monitoring campaign in the four investigated rooms. Points (dashed lines) represent kitchens and triangles (full lines) bedrooms; black symbols and lines correspond to flat 1; light grey symbols and lines correspond to flat 2. S2a: winter campaign, 28 January to 15 February 2020; S2b: spring campaign, 23 May to 06 June 2020; S2c: summer campaign, 29 June to 13 July 2020.
